# Underdiagnosis of frontotemporal lobar degeneration in Brazil

**DOI:** 10.1590/S1980-57642008DN10400006

**Published:** 2007

**Authors:** Valéria Santoro Bahia

**Affiliations:** MD, PhD Brazil. Behavioral and Cognitive Neurology Unit, Department of Neurology, Hospital das Clínicas, University of São Paulo School of Medicine, São Paulo.

**Keywords:** frontotemporal lobar degeneration, frontotemporal dementia, Alzheimer’s disease, semantic dementia, primary progressive aphasia, degeneração lobar fronto-temporal, demência fronto-temporal, doença de Alzheimer, demência semântica, afasia progressiva primária

## Abstract

Frontotemporal lobar degeneration (FTLD) is a progressive neurodegenerative disorder that involves the frontal and anterior temporal lobes. It is characterized by prominent behavioral and language disorders. There are three variants of FTLD: frontotemporal dementia (FTD), semantic dementia (SD) and progressive non-fluent aphasia (PNFA). FTLD may be mistaken for AD (Alzheimer’s disease) or psychiatric disorders especially in the early stages.

**Objectives:**

To describe the demographic characteristics of patients with FTLD, assessed at the Behavioral and Cognitive Neurology Unit, and to show that FTLD is commonly clinically under-diagnosed.

**Methods:**

All patients diagnosed with FTLD (Consensus Criteria for FTLD), and who were seen at the Behavioral and Cognitive Neurology Unit of Hospital das Clínicas, in São Paulo, Brazil from January 2004 to August 2007 were included in the analyses.

**Results:**

Sixteen patients with FTLD (11 women) were included in this study. There were 12 patients with FTD, two with PNFA and two with SD. The mean duration of the illness until diagnosis of FTLD was 4.1±2.3 years, ranging from one to seven years. Nine patients had been initially seen by psychiatrists, five by neurologists, and two by general physicians. The first diagnosis was psychiatric disorder in six patients, AD in four, dementia in two, FTD in two, and stroke and hydrocephalus for one patient each.

**Conclusion:**

The diagnosis of FTLD can be difficult and many patients may be misdiagnosed in Brazil, especially in the initial stages. Educational programs on FTLD for the medical community are warranted.

## Abstract (pt)

Degeneração Lobar Fronto-temporal (DLFT) é uma doença neurodegenerativa progressiva que acomete lobos frontais e temporais. Caracteriza-se pela predominância de distúrbios comportamentais e de linguagem. Há três variantes da DLFT: demência fronto-temporal (DFT), demência semântica (DS) e afasia progressiva não fluente (APNF). Pacientes com DLFT podem ser diagnosticados erroneamente, principalmente nas fases iniciais.

**Objetivos:**

Descrever as características demográficas dos pacientes avaliados no Grupo de Neurologia Cognitiva e do Comportamento e mostrar que os casos de DLFT são freqüentemente diagnosticados erroneamente em fase inicial.

**Métodos:**

Avaliamos todos os indivíduos com o diagnóstico de DLFT, (Critérios de Consenso de DLFT) atendidos no Grupo de Neurologia Cognitiva e do Comportamento do Hospital das Clínicas, São Paulo, Brasil, entre Janeiro de 2004 e Agosto de 2007.

**Resultados:**

Foram incluídos 16 pacientes com DLFT (11 mulheres). Doze pacientes tiveram o diagnóstico de DFT, dois de DS e dois APNF. A média de duração da doença até o diagnóstico de DLFT foi de 4,1±2,3 anos (variando de um a sete anos). Nove pacientes foram avaliados, no início da doença, por psiquiatras, cinco por neurologistas e dois por clínicos gerais. O primeiro diagnóstico foi de distúrbio psiquiátrico em seis pacientes, DA em quatro pacientes, demência em dois, DFT em dois, acidente vascular cerebral em um e hidrocefalia em um paciente.

**Conclusão:**

O diagnóstico de DLFT pode ser difícil e muitos pacientes, principalmente em fases iniciais, podem estar sendo diagnosticados erroneamente no Brasil. Há a necessidade de maior informação sobre a doença na comunidade médica.

Frontotemporal lobar degeneration (FTLD) is a progressive neurodegenerative disorder that involves the frontal and temporal lobes. It is characterized by prominent and gradual behavioral and language disorders, whereas memory is relatively preserved.^1,2^

FTLD is the second most common cause of degenerative dementia after Alzheimer’s disease (AD) in the presenile period (45-65 years) having a low prevalence in the elderly.^3-6^ In a study of the prevalence of dementia in a community-dwelling population, aged 65 years or more living in Catanduva, Brazil only 2.5% of the demented patients had the diagnosis of FTD.^7^

Neary et al.^1^ distinguished three variants of FTLD with hallmark sets of presenting symptoms and regional patterns of atrophy: the frontal variant of frontotemporal dementia (FTD), semantic dementia (SD) and progressive non-fluent aphasia (PNFA).

FTD is the most common clinical pr esentation, and accounts for approximately half of all FTLD diagnoses. The characteristic features include loss of insight, disinhibition, impulsivity, apathy, reduced empathy for others, poor self care, stereotypic behavior, emotional blunting, and changes in eating patterns.^5,8^

Two other clinical subtypes of FTLD have been characterized for the most prominent symptoms of the language dysfunction. PNFA is a disorder of expressive language, including nonfluent spontaneous speech and word retrieval difficulties with phonological and grammatical errors. SD is a disorder characterized by progressive loss of knowledge on words and objects in which fluency of speech is maintained, along with agnosia for faces and objects. PNFA and particularly SD, tend to aggregate behavioral abnormalities over the course of the disease.^9-11^

Diagnosis of FTD, PNFA and SD are not straightforward, often being mistaken for AD or psychiatry disorders, especially in the early stages.^12-15^

The objective of this study was to describe the demographic characteristics of the patients with FTLD assessed at the Behavioral and Cognitive Neurology Unit and to show that FTLD is usually clinically under-diagnosed.

## Methods

All patients who fulfilled consensus criteria^1^ for FTLD (FTD, SD, APNF) and seen from January 2004 to August 2007 at the Behavioral and Cognitive Neurology Unit of Hospital das Clínicas, in São Paulo, Brazil were included in the study, where all subjects were evaluated by neurologists and neuropsychologists.

The diagnosis was based on anamneses, neurological examination, and neuropsychological assessment that included the Brief Cognitive Battery,^16^ Mattis Dementia Rating Scale (DRS),^17^ Frontal Assessment Battery (FAB).^18^ Activities of daily living were evaluated using the Functional Activities Questionnaire (FAQ).^19^

All patients underwent structural neuroimaging (CT or MRI) and functional SPECT imaging along with a battery of routine screening blood tests.

The age at onset was defined as the age at which the first symptom compatible with the diagnosis FTLD appeared as reported by the principal informant, while the duration of illness was defined by the interval between age at onset and age on first assessment. Education was considered as the number of years of formal education.

Descriptive statistics analyses were conducted using BioEstat 3.0 software.

## Results

Sixteen patients, 5 men and 11 women, with mean age of 57.6±6.7 years, mean age at onset of 53.6±7.8 years (ranging from 36 to 63 years) and mean years of schooling of 8.0±6.2were included. Overall, FTD was the most common diagnosis, accounting for 12 patients, followed by SD and PNFA with two patients each.

Five patients were not submitted to neuropsychological assessment due to severity of the illness.

The mean MMSE score was 13.7±9.9and for FAQ was 22.7±7.5.

The mean interval between the first symptom and the first structural neuroimage was 2.6±2.4 years, and for the first SPECT was 3.0±2.4 years. The structural neuroimages (CT or MRI) were compatible with the diagnosis in 87.5% of the cases, and the SPECT in the 81.3%. Only one patient, with a very typical clinical picture of FTD presented diffuse atrophy on MRI and diffuse cortical irregularities on SPECT.

The duration of illness until diagnosis of FTLD was 4.1±2.3 years.

**Figure 1:**
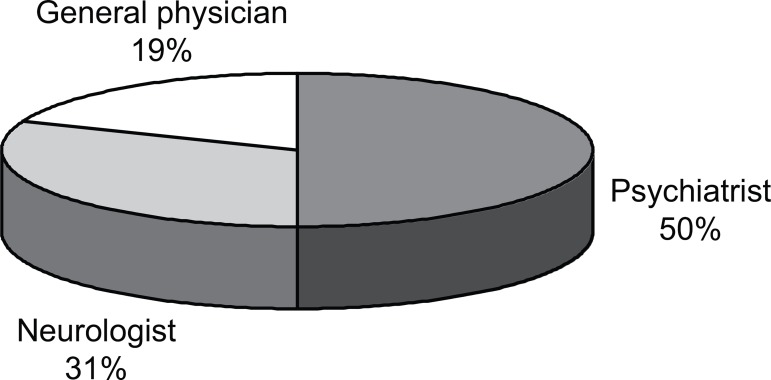
First specialist to see patients.

Nine patients had been initially seen by psychiatrists, five by neurologists and two by general physicians (Figure 1).

**Figure 2:**
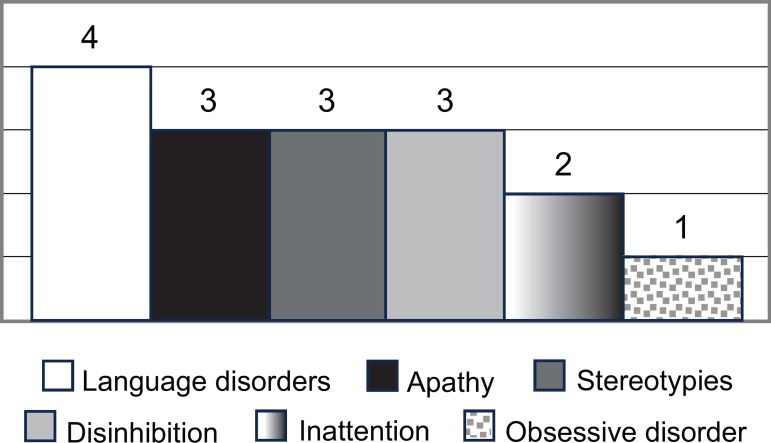
First symptom of the disease.

The first symptom was language dysfunction in four patients, apathy in three, stereotypic behavior in three, disinhibition in three, inattention in two, and obsessive symptoms in one patient (Figure 2).

**Figure 3:**
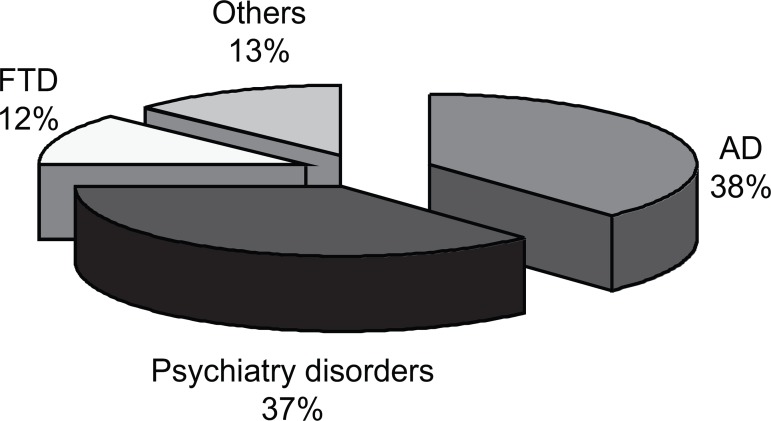
First diagnosis of the patients. AD: Alzheimer’s disease; FTP: frontotemporal dementia.

The first diagnosis was psychiatric disorder in six patients, AD in four patients, dementia in two patients, FTD in two, and stroke and hydrocephalus for one patient each (Figure 3).

## Discussion

In our sample, all patients presented early onset of the disease and 87.5% of the patients were misdiagnosed for 4.1±2.3 years after the first symptoms. Structural and functional neuroimaging was performed only after 2.6±2.4 years and 3.0 ± 2.4 years, respectively. Consequently, these patients had delayed diagnoses and risked inappropriate therapy.

In general, the earliest manifestation of FTLD are behavioral changes, executive dysfunctions or language disorders while for AD there is a predominance of impaired episodic memory and visuospacial skills.^12^

Some FTLD patients have memory complaints, usually related to the disexecutive syndrome or to word finding difficulties from language dysfunction,^12,13^ although there are some evidences of hippocampal atrophy in SD and FTD patients.^20-22^

Despite these differences in neuropsychological profiles, there are evidences that clinical differentiation between FTLD and AD remains poor. A study using the NINCDS-ADRDA (National Institute for Communicative Disorders and Stroke - Alzheimer’s Disease and Related Disorders Association) for a differential diagnosis between probable AD and FTD showed high sensitivity (0.93) but low specificity (0.23). In the cited study, twenty patients with pathological confirmation of FTD fulfilled criteria for AD.^14^

Rosen et al.^12^ performed a retrospective study comparing the initial presentation of 30 FTLD patients and 30 AD patients, and showed that 63% of the patients with FTLD were correctly identified at the initial clinical evaluation using consensus criteria.^1^ Five clinical features, including the presence of social conduct disorders, hyperorality, and akinesia, as well as absence of amnesia and absence of perceptual disorders, allowed the identification of 93% of the FTLD and 97% of the AD patients.

Mendez et al.^15^ compared the sensitivities and specificities of different diagnostic methods while investigating 134 patients with suspected FTD. The clinical diagnosis based on the consensus criteria^1^ attained sensitivity of 36.5% and specificity of 100%, being 63.5% and 70.4% for structural neuroimage, and 90.5% and 74.6% for SPECT/PET scan, respectively. They demonstrated that neuropsychological results did not distinguish FTD from other causes of dementia, but the pattern of progression helped to establish the diagnosis after two years of follow up.

In our sample, after language disorders, which are the most frequent symptoms in patients with progressive aphasia (SD and PNFA), aberrant motor behavior (perseverative and compulsive behaviors), apathy and disinhibition were the most frequent first symptoms. These symptoms occur more frequently in FTLD than AD patients.^23-25^ Due to this, there are studies that advocate the use of behavioral scales in differential diagnosis between FTD and AD, and are considered better than neuropsychological tests.^26,27^

Physicians continue to experience difficulty in diagnosing early FTD because patients with behavioral changes are often referred to psychiatry services with suspected diagnosis of depression, personality disorders or schizophrenia, in spite of the low prevalence of psychotic symptoms in FTD.^28^

Schizophrenic patients may show a wide range of cognitive impairments, most prominently deficits in memory, executive functions,^29^ disorientation and impaired daily living skills.^30^ There is a lack of structured psychiatry assessment in differentiating psychiatric diseases from FTLD.^31^ There are few reports of cases of FTLD misdiagnosed as schizophrenia but in all such cases the structural neuroimaging exam confirmed the diagnosis of FTLD although carried out in delayed phase.^32-34^

Our study has some methodological limitations. First, it was a retrospective study, and in some cases specific features may not have been reported by the caregiver. Second, the study lacked autopsy confirmation.

In conclusion, many patients with FTLD may be misdiagnosed, especially in the initial stages. FTLD should be distinguished from psychiatric disorders, AD and others dementias, because there are differences in treatment and prognosis. This differentiation can be difficult and demands an astute qualitative analysis of behaviors and neuropsychological test performances in conjunction with analyses of neuroimaging exams together with information from relatives. Provision of more information on FTLD to the medical community in Brazil is warranted.
